# Machine learning-informed liquid-liquid phase separation for personalized breast cancer treatment assessment

**DOI:** 10.3389/fimmu.2024.1485123

**Published:** 2024-11-19

**Authors:** Tao Wang, Shu Wang, Zhuolin Li, Jie Xie, Huan Chen, Jing Hou

**Affiliations:** ^1^ Research Laboratory Center, Guizhou Provincial People’s Hospital, Guiyang, China; ^2^ Department of Breast Surgery, Guizhou Provincial People’s Hospital, Guiyang, China

**Keywords:** breast cancer, liquid-liquid phase separation, machine learning, immunotherapy, methotrexate

## Abstract

**Background:**

Breast cancer, characterized by its heterogeneity, is a leading cause of mortality among women. The study aims to develop a Machine Learning-Derived Liquid-Liquid Phase Separation (MDLS) model to enhance the prognostic accuracy and personalized treatment strategies for breast cancer patients.

**Methods:**

The study employed ten machine learning algorithms to construct 108 algorithm combinations for the MDLS model. The robustness of the model was evaluated using multi-omics and single-cell data across 14 breast cancer cohorts, involving 9,723 patients. Genetic mutation, copy number alterations, and single-cell RNA sequencing were analyzed to understand the molecular mechanisms and predictive capabilities of the MDLS model. Immunotherapy targets were predicted by evaluating immune cell infiltration and immune checkpoint expression. Chemotherapy targets were identified through correlation analysis and drug responsiveness prediction.

**Results:**

The MDLS model demonstrated superior prognostic power, with a mean C-index of 0.649, outperforming 69 published signatures across ten cohorts. High-MDLS patients exhibited higher tumor mutation burden and distinct genomic alterations, including significant gene amplifications and deletions. Single-cell analysis revealed higher MDLS activity in tumor-aneuploid cells and identified key regulatory factors involved in MDLS progression. Cell-cell communication analysis indicated stronger interactions in high-MDLS groups, and immunotherapy response evaluation showed better outcomes for low-MDLS patients.

**Conclusion:**

The MDLS model offers a robust and precise tool for predicting breast cancer prognosis and tailoring personalized treatment strategies. Its integration of multi-omics and machine learning highlights its potential clinical applications, particularly in improving the effectiveness of immunotherapy and identifying therapeutic targets for high-MDLS patients.

## Introduction

Breast cancer (BC) is a heterogeneous disease and is the most common cancer in women. Breast cancer morbidity and mortality are separately reported as 11.7% and 6.9%, respectively, by GLOBCAN, 2020 (1). In women, it remains one of the most common causes of cancer-related death (2). Therefore, improving the efficiency of early diagnosis to identify breast cancer more reliably has become a research hotspot worthy of attention. Developing personalized treatment strategies for the clinic is crucial, and one approach involves creating predictive models to aid in the early detection and diagnosis of breast cancer.

Despite significant advances in developing predictive models for breast cancer, the outcomes remain suboptimal. Breast cancer incidence is believed to be closely associated with transcriptional dysregulation or genetic mutations (3). Recent insights into the biophysical behavior of cells highlight the role of liquid-liquid phase separation (LLPS), involving multivalent interactions among RNA, proteins, and other molecular structures. These interactions often result in the formation of droplet-like units known as membranelles, which exhibit distinct liquid-like properties (4, 5). These organelles maintain a stable internal environment by dynamically exchanging components with surrounding cellular structures (6, 7). The concept of protein and nucleic acid LLPS has emerged as a new research paradigm due to its significant impact on cellular activity and its underlying mechanisms (8). LLPS plays a pivotal role in various biological processes, including chromatin organization, transcription, DNA damage response, autophagy, X chromosome inactivation, and even tumor growth and metastasis (9–11). For example, the long non-coding RNA (lncRNA) SNHG9 has been shown to induce LLPS in the kinase LATS1, promoting the growth of breast cancer cells (5).

The role of autophagy-related genes, immune genes, and other factors in predicting tumor prognosis has been extensively studied (12, 13). However, few investigations have explored the potential of LLPS-related genes in this context. Notably, recent studies have highlighted the prognostic significance of LLPS-related genes in cancers such as lung squamous cell carcinoma, where they have been incorporated into prognostic models (14). In our research on BC progression, we conducted a comprehensive analysis to elucidate the importance of LLPS. Leveraging single-cell sequencing techniques, we evaluated LLPS activity across various immune cell types. Machine learning algorithms were then employed to identify LLPS genes associated with BC prognosis, allowing us to construct predictive models (15, 16). These models demonstrated the efficacy of LLPS in predicting BC patient outcomes, immune status, responsiveness to immune checkpoint inhibitors (ICIs) and chemotherapy, as well as in identifying potential therapeutic targets and drugs. Through rigorous evaluations, LLPS emerged as a promising tool for precise prognostication and treatment stratification in BC patients.

## Methods

### Data acquisition

We conducted a retrospective collection of 14 distinct breast cancer cohorts sourced from The Cancer Genome Atlas (TCGA), Gene Expression Omnibus (GEO), and Metabric (17). These cohorts comprised samples with comprehensive survival data, which were subsequently utilized for in-depth analysis. In total, our study encompassed 22,162 patients across the 14 cohorts for the purpose of prognostic assessment. The distribution of patients across cohorts was as follows: TCGA-BRCA (n = 1076), GSE202203 (n = 3206), GSE96058 (n = 3409), GSE20685 (n = 327), GSE86166 (n = 330), GSE1456 (n = 159), GSE21653 (n = 244), GSE7390 (n = 198), GSE11121 (n = 200), GSE6532 (n = 87), GSE88770 (n = 108), GSE48391 (n = 81), GSE131769 (n = 298) and Metabric (n = 1747).

### Machine learning derived LLPS signature

To develop a LLPS signature specific to breast cancer, we adopted the methodology outlined by our previous research (18). Our approach entailed the utilization of ten diverse computational techniques: Random Survival Forest (RSF), Least Absolute Shrinkage and Selection Operator (LASSO), Gradient Boosting Machine (GBM), Survival Support Vector Machine (Survival-SVM), Supervised Principal Component (SuperPC), Ridge Regression, Partial Least Squares Cox Regression (plsRcox), CoxBoost, Stepwise Cox regression, and Elastic Net (Enet). Notably, RSF, LASSO, CoxBoost, and Stepwise Cox were selected for their capacity to diminish dimensionality and identify pertinent variables.

We then combined the machine learning algorithms into 108 unique combinations to construct a robust MDLS model. Each combination was trained on multi-omics data, with the average Concordance Index (C-index) used as the performance metric to identify the most predictive model. Through iterative cross-validation, we systematically evaluated the predictive accuracy of each algorithm combination across multiple breast cancer cohorts.

From the analysis, we identified the most consistent and predictive model. Four LLPS-related genes (POP1, TUBA1C, RACGAP1, and PLK1) were selected as key features based on their prognostic value, as determined by univariate Cox regression analysis. These genes formed the foundation of the final MDLS signature, which was optimized to predict patient outcomes in breast cancer.

To define the high- and low-MDLS groups, we used the surv_cutpoint function from the “survminer” R package. This function calculated the optimal cutoff value that maximally separated patients into high- and low-risk groups based on their survival data. Patients with MDLS risk scores above the cutoff were classified as the high-MDLS group, indicating a higher risk profile, while those with risk scores below the cutoff were classified into the low-MDLS group.

The performance of the MDLS signature was validated across 14 independent breast cancer cohorts, incorporating both bulk tumor and single-cell RNA sequencing data. In total, the cohorts involved over 9,723 breast cancer patients, ensuring a comprehensive evaluation. Additionally, we compared the MDLS signature with 69 published breast cancer signatures, and the MDLS demonstrated superior prognostic power across the cohorts.

### Genomic alteration analysis

To delineate genetic disparities between the two MDLS groups, we conducted an analysis of both genetic mutation levels and Copy Number Alterations (CNA) utilizing the TCGA-BRCA database.

The Tumor Mutation Burden (TMB) of both high- and low MDLS breast cancer patients was extracted from the raw mutation file. Utilizing the maftools landscape, we depicted the most frequently mutated genes (mutation rate > 5%). Additionally, patient-specific mutational signatures were identified using the deconstructSigs package (19). Notably, we emphasized four prominent mutational signatures (SBS3, SBS1, SB12, SBS11) within the TCGA-BRCA dataset that displayed heightened mutation frequencies. We identified the five most common regions of amplification and deletion and specifically highlighted the four predominant genes in chromosomal regions 8q24.21 and 5q11.2.

### Single-cell data processing

For the preparation of the dataset for single-cell RNA sequencing analysis, we employed Seurat (v4.0) to process the data extracted from GSE161529 (20). This process involved filtering out genes with no expression and retaining those with nonzero expression levels. Normalization of the expression matrix was performed using Seurat’s “SCTransform” function. Dimensionality reduction of the dataset was achieved through (principal component analysis) PCA and UMAP reductions. To identify distinct cellular groupings, Seurat’s “FindNeighbors” and “FindClusters” functions were utilized. To ensure dataset integrity and reliability, the DoubletFinder package was employed to eliminate potential doublets (21). Cells failing to meet defined quality standards, such as exhibiting mitochondrial gene content exceeding 15% or containing fewer than 500 genes, were excluded. Through stringent quality control measures, a total of 47,784 cells were retained for subsequent analysis. Cell types were determined by manual annotation based on the presence of established marker genes.

### Inference of regulons and their activity

In our investigation, we adopted the Single-Cell rEgulatory Network Inference (SCENIC) approach to construct gene regulatory networks (GRNs) from single-cell RNA sequencing data. SCENIC involves a three-step process: initially, it identifies co-expression modules between transcription factors (TFs) and their potential target genes. Subsequently, for each module, it identifies the direct target genes, prioritizing those enriched with the motif of the associated TF. A regulon is then defined, comprising a TF and its direct targets. Finally, the regulatory activity score (RAS) is computed for each cell by assessing the area under the recovery curve.

While the conventional SCENIC protocol encounters challenges with scalability for extensive datasets and is susceptible to variations in sequencing depth, we introduced a modification to enhance both scalability and robustness. This involved partitioning the data into metacells before applying SCENIC to these gene expression profiles (22). This adjustment significantly enhances data quality and reduces computational demands, representing a notable advancement in the application of SCENIC to single-cell RNA-seq data analysis.

### Regulon clustering

We implement a comprehensive computational approach to elucidate the regulatory interplay between transcription factors (TFs) and their corresponding target genes, with a particular focus on TF clustering. Initially, the method entails filtering TF-target interaction data to isolate pairs that exceed a predefined significance threshold (>1), ensuring prioritization of regulatory interactions of utmost relevance. Subsequent analysis aims to identify key regulatory TFs by assessing the extent of their target gene regulation, highlighting them as central nodes within the regulatory network for in-depth investigation.

To visually represent the complex network of TF-target interactions, an undirected graph model is constructed. The spatial arrangement of this graph is refined using a force-directed algorithm to intuitively depict the network’s architecture, emphasizing the interplay between TFs and their targets. Additionally, to enhance comprehension of the network’s structure, the Leiden algorithm is applied for community detection. This process unveils the modular configuration of TFs based on their regulatory interconnections, assigning each TF to a distinct cluster. This facilitates a nuanced analysis of the regulatory landscape, enabling insights into the functional organization of TFs within the network.

### Cell-cell communication analysis

Using the “CellChat” R package, CellChat objects were generated based on the UMI count matrices for each respective group (23). The “CellChatDB.human” database served as the reference for ligand-receptor interactions. Interpreting intercellular communication was executed using the default settings provided by the package. To assess and compare interaction counts and intensities, CellChat objects from each group were merged using the “mergeCellChat” function. Variations in the number and intensity of interactions among specific cell types across different groups were visualized using the “netVisual_diffInteraction” function. Additionally, changes in signaling pathways were identified with the “rankNet” function, and the distribution of signaling gene expression among the groups was illustrated using both the “netVisual_bubble” and “netVisual_aggregate” functions.

Furthermore, we applied the NicheNet package to analyze intercellular communication from the perspective of ligand activity and the expression patterns of specific downstream targets regulated by these key ligands (24). This approach enables a detailed understanding of the signaling processes underlying interactions between different cell types, leveraging information about ligand-target relationships to infer communication pathways within the cellular microenvironment.

### Evaluation of TME disparities and immunotherapy response

In our endeavor to comprehensively and accurately assess immune cell infiltration levels, we conducted an analysis of adverse infiltrated immune cells across multiple algorithms, including MCPcounter, EPIC, xCell, CIBERSORT, quanTIseq, and TIMER, among patients stratified by the MDLS (25–31). Additionally, to depict the immune landscape and architecture within the tumor microenvironment (TME) with precision, we evaluated the ESTIMATE and TIDE indices. These measures provide crucial insights into the potential for immunotherapy and offer prognostic implications for breast cancer patients.

Furthermore, we quantified immune checkpoints, which serve as indicators of the immune state and offer preliminary predictions of patient responsiveness to immune checkpoint inhibitor (ICI) therapy. This comprehensive approach to evaluating the immune profile within the TME is essential for advancing personalized medicine and refining treatment strategies for breast cancer patients.

### Determination of therapeutic targets and drugs for high MDLS patients

Our methodology for identifying therapeutic targets and drugs for high MDLS patients commenced by filtering out duplicate compounds from the Drug Repurposing Hub, resulting in a refined list of 6,125 compounds. The selection of therapeutic targets associated with breast cancer outcomes was established through Spearman correlation analysis. Specifically, we assessed the relationship between the MDLS and gene expression levels, selecting genes with a correlation coefficient greater than 0.3 and a P-value less than 0.05. Additionally, genes demonstrating a correlation coefficient below -0.3 and a P-value below 0.05 were identified as linked to poor prognosis. The significance of these genes was further evaluated by examining the relationship between CERES scores from the Cancer Cell Line Encyclopedia (CCLE) and risk scores (32).

To enhance predictions regarding drug responsiveness, we utilized data from the Cancer Therapeutics Response Portal (CTRP) and the PRISM project, both of which offer extensive drug screening and molecular data across diverse cancer cell lines. Differential expression analysis was conducted between bulk samples and cell lines. Subsequently, the pRRophetic package was employed to implement a ridge regression model for predicting drug response. This model, trained using expression data and drug response metrics from solid Cancer Cell Lines (CCLs), demonstrated excellent predictive accuracy, validated through 10-fold cross-validation (33).

Furthermore, to identify the most promising therapeutic drugs for breast cancer, Connectivity Map (CMap) analysis was performed. This entailed comparing gene expression profiles across different risk subgroups and submitting the top 300 genes (comprising 150 up-regulated and 150 down-regulated genes) to the CMap website. Interestingly, a negative CMap score indicated a higher therapeutic potential against breast cancer, suggesting an inverse relationship between the CMap score and a compound’s effectiveness as a potential treatment.

### Patient stratification

To assess gene expression in breast cancer specimens, RNA extraction was performed using TRIzol reagent (Invitrogen, Carlsbad, CA, USA), followed by cDNA synthesis and quantitative reverse transcription PCR (qRT-PCR) using GoScript reverse transcriptase and Master Mix (Promega), following the manufacturer’s guidelines. The CFX96 Touch Real-Time PCR Detection System (BioRad, Hercules, CA, USA) was employed for data acquisition. Gene expression quantification was executed using the 2^-ΔΔCq^ method, with GAPDH utilized as the normalization control. Subsequently, patients were categorized based on their gene expression profiles, utilizing a predefined formula derived from the MDLS. This stratification played a crucial role in identifying patients with distinct risk profiles, thereby enabling tailored therapeutic interventions.

### Immunohistochemistry experiment

Tissue samples were obtained from 30 breast cancer patients undergoing surgery at Guizhou Provincial People’s Hospital (**Supplementary Table S1**). These samples underwent Hematoxylin and Eosin (H&E) staining using established protocols. The diagnosis was independently confirmed by two pathologists.

For the immunohistochemistry (IHC) analysis, procedures for paraffin-embedded samples were followed, as outlined in previous studies (34, 35). Protein expression levels were assessed independently by two pathologists, adhering to standardized protocols and scoring systems consistent with methodologies from prior research (35).

### Statistical analysis

Statistical analyses were performed to assess the differences between the high- and low-MDLS groups. Continuous variables were compared using the unpaired Student’s t-test or Mann-Whitney U test, depending on the distribution of the data. Chi-square test or Fisher’s exact test was employed to compare categorical variables. Pearson’s correlation test was used to evaluate correlations between gene expression levels and clinical variables.

Survival outcomes were analyzed using Kaplan-Meier analysis, and differences between groups were evaluated with the log-rank test. Cox proportional hazards regression models were used for both univariate and multivariate survival analyses to identify independent prognostic factors.

All statistical analyses were conducted using R (version 4.0.5), and the survminer R package. Statistical significance was defined as a p-value of less than 0.05, with differences considered significant at *p < 0.05, **p < 0.01, or ***p < 0.001.

## Results

### Construction of a LLPS signature based on machine learning

**Figure 1:**
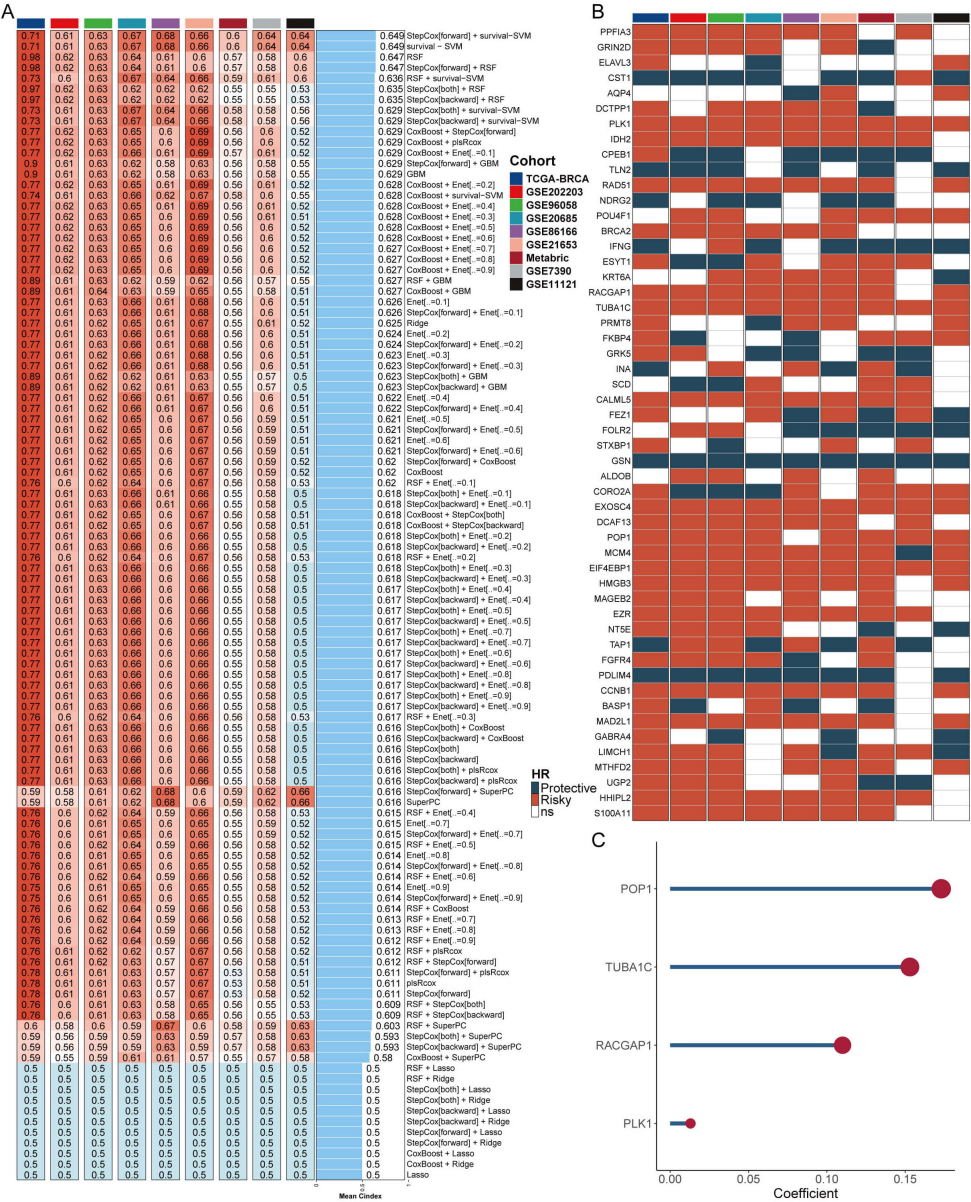
Construction of a LLPS signature based on machine learning. **(A)** The C-indexes of 108 machine-learning algorithm combinations in the nine testing cohorts. **(B)** Key LLPS genes associated with breast cancer prognosis. **(C)** Genes to construct the MDLS.

From the DrLLPS database (36), we collected LLPS genes to conduct differential expression analyses in TCGA-BRCA between tumor and normal tissues. A ten-fold cross-validation process was applied to construct prediction models using 108 algorithm combinations and calculated the mean C-index of each algorithm in the training cohort (TCGA-BRCA) and 8 external cohorts. The combination of StepCox[forward] and survival-SVM with the highest mean C-index (0.649) was selected as the final model (**Figure 1A**). We assessed the prognostic value of these LLPS genes based on univariate Cox regression and calculated the Hazard ratio (HR) for these genes in the nine enrolled cohort (**Figure 1B**). As shown in **Figure 1C**, four positively correlated risk factor genes (POP1, TUBA1C, RACGAP1, PLK1) were screened to build the machine learning derived LLPS signature (MDLS). The findings revealed that our model successfully differentiated between high- and low-MDLS patients, suggesting that MDLS can offer valuable reference information for predicting the survival of breast cancer patients (**Supplementary Figure S1**).

### Evaluation of MDLS with 69 published signatures in BC

**Figure 2:**
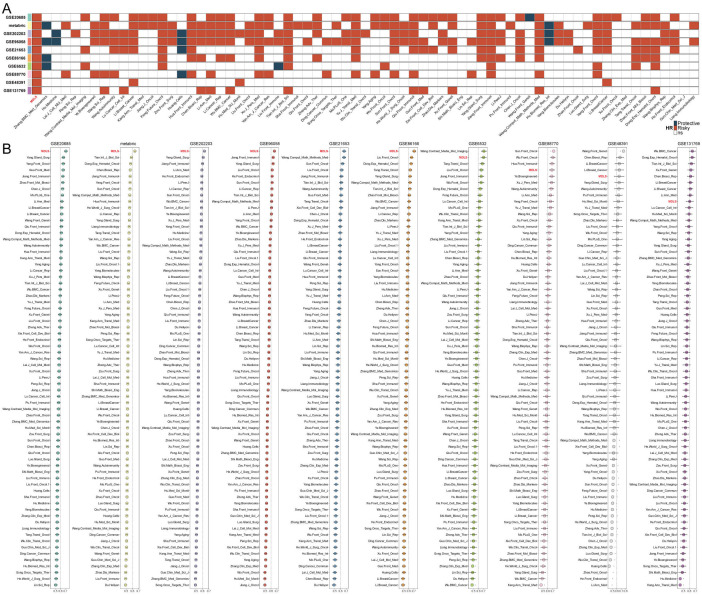
Evaluation of MDLS with 69 published signatures in BC. **(A)** The stability of the MDLS was compared with 69 published models. **(B)** C-indices of MDLS and 69 published signatures in 10 datasets.

Univariate and multivariate Cox analysis showed that MDLS was an independent risk factor compared with other clinical indicators (**Supplementary Figure S2A**). The nomogram consisting of MDLS, stage and age was resorted to accurately predict the OS of BC patients at distinctive times, and the prediction effect of MDLS is better (**Supplementary Figures S2B–F**). The kernel-smoothing hazard plot show that high-MDLS patients had a poorer outcome and higher recurrence frequency than low-MDLS patients (**Supplementary Figure S2G**). To evaluate the stability of the predictive model of the MDLS, 68 published signatures in BC were manually collected and assessed in 10 independent cohorts. We demonstrated that only the MDLS had consistent statistical significance across all cohorts (**Figure 2A**). We compared the predictive power of MDLS with those 69 features across 10 cohorts using the C-index (**Figure 2B**). Our model showed significantly better accuracy than the others in almost all cohorts (ranking first in six cohorts, second in one cohort, fourth in one cohort, fifth in one cohort, and ninth in one cohort), revealing the stability of the MDLS.

### Genetic alteration landscape of MDLS

**Figure 3:**
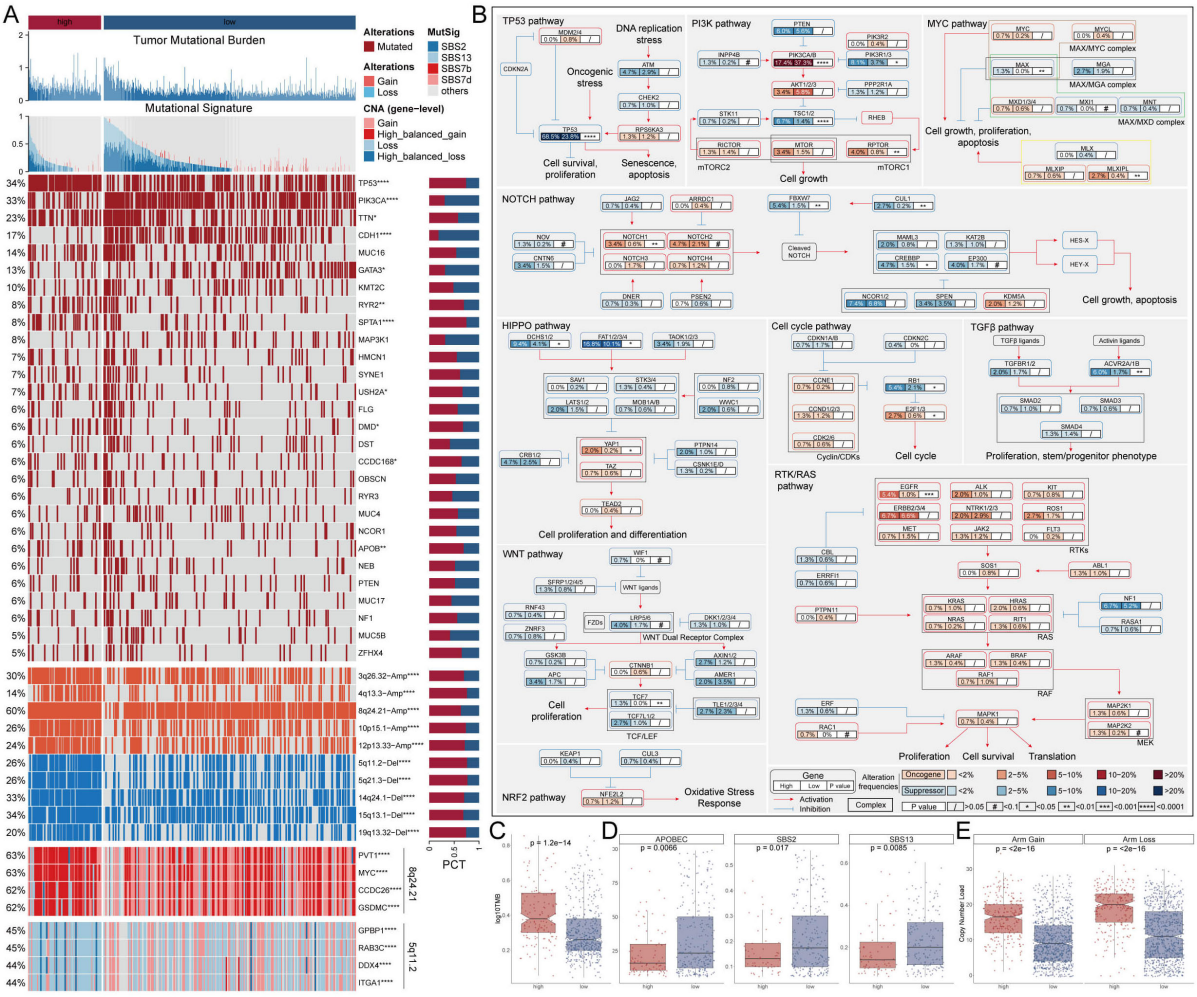
Genetic alteration landscape of MDLS. **(A)** Genomic alteration landscape based on MDLS. **(B)** Detailed comparison of 10 oncogenic signaling pathways between groups with high and low MDLS. **(C)** Comparison of TMB. **(D)** Mutant signatures were shown for SBS2 and SBS13, SBS7b and SBS7d. **(E)** Comparison of CNA at the chromosome arm level. ^*^P<0.05, ^**^P<0.01, ^****^P<0.0001.

To explore genomic heterogeneity between high- and low-MDLS groups, we analyzed gene mutation and copy number variation across the groups (**Figure 3A**). We observed high tumor mutation burden (TMB) in the high-MDLS patients (**Figures 3A, C**). Combining the 10 oncogenic signaling pathways in the TCGA database, we found that a total of 12 classical tumor suppressor genes and 6 oncogenes were mutated more frequently in the high-MDLS group (**Figure 3B**). Moreover, mutational signature analysis shows that the frequencies of SBS2, SBS13, SBS7b, SBS7d were significantly higher in low-MDLS (**Figures 3A, D**). The number of classical mutations (SBS2 and SBS13) in APOBEC3 was examined, the results showed the same trend (**Figure 3D**). Next, we delved deeper into the CNA scenery of the two groups. Compared to the low-MDLS group, the high-MDLS group owned evidently higher deletion or amplification in the chromosome arm levels, like the amplification of 3q26.32, 4q13.3, 8q24.21, 10p15.1, 12p13.33, and the deletion of 5q11.2, 5q21.3, 14q24.1, 15q13.1, 19q13.32 (**Figures 3A, E**). At the gene level, there was significant gene amplification on chromosome 8q24.21 (PVT1, MYC, CCDC26, GSDMC) and significant gene deletion on chromosome 5q11.2 (GPBP1, RAB3C, DDX4, ITGA1) in the high-MDLS group (**Figure 3A**). In conclusion, tumor suppressor gene deletion and oncogene amplification in the high-MDLS group may be one of the reasons for the poor prognosis.

### Understanding the biological mechanisms of MDLS at the single-cell level

**Figure 4:**
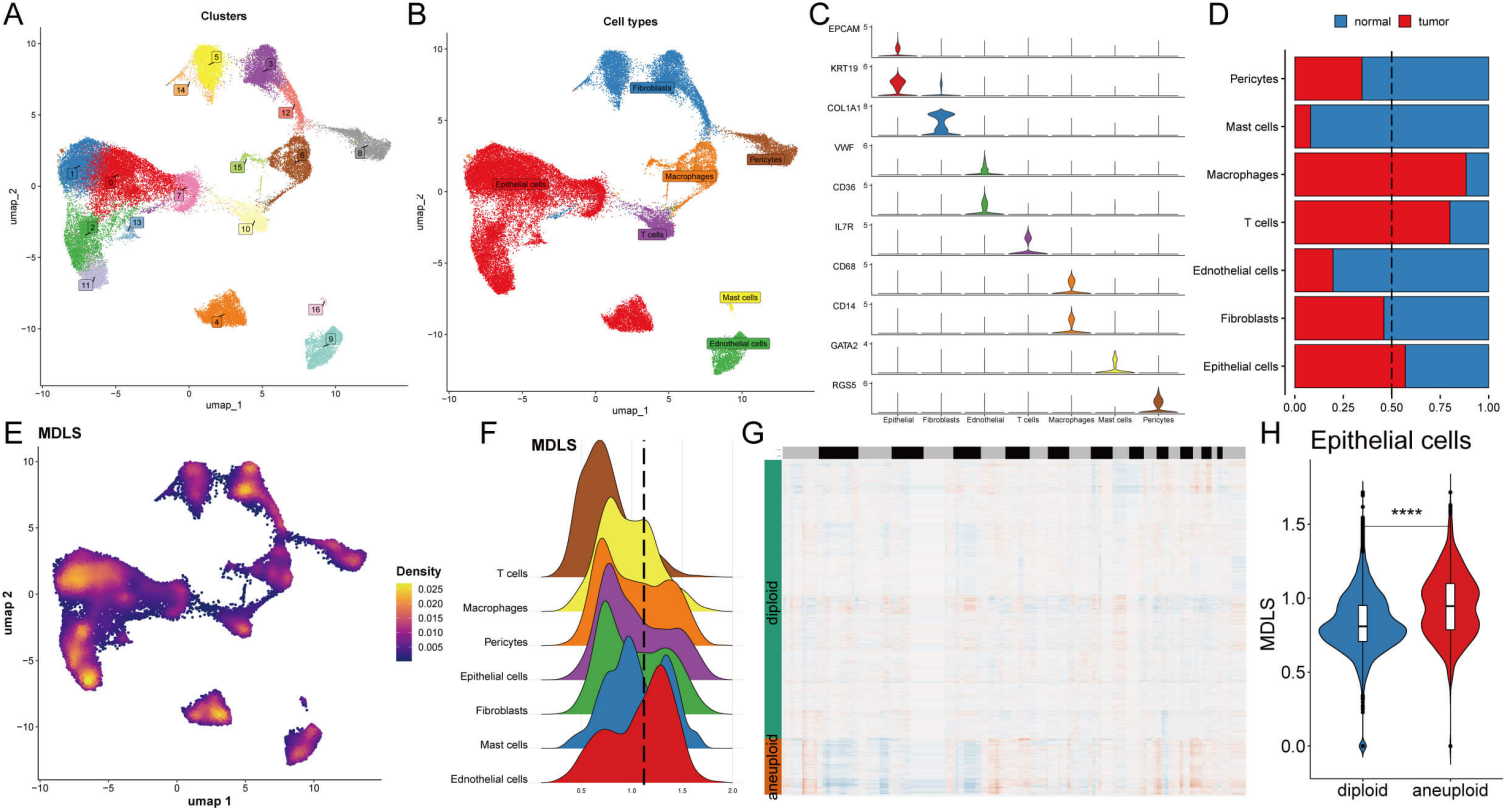
Understanding the biological mechanisms of MDLS at the single-cell level. **(A)** The distribution of 17 cell clusters. **(B)** The distribution of 7 cell types. **(C)** The representative markers in 7 cell types. **(D)** The proportion of 7 types of cells in normal and BC tissues. **(E)** Specific single cell distribution map in the MDLS value. **(F)** The distribution of MDLS value across various cell types. **(G)** CopyKat algorithm analyzed the distribution of diploid and aneuploid cells. **(H)** Comparison of the MDLS score between diploid and aneuploid cells within the epithelial cell population. ^****^P<0.0001.

We selected 8 patients with breast cancer for further evaluation of MDLS, including 4 tumor tissues and 4 normal tissues (**Supplementary Figures S3A, B**). The cells were divided into 17 clusters and 7 cell types (**Figures 4A, B**). Statistical analysis was conducted to determine the overall number and proportion of these seven types of cells in the bodies of these eight tumor patients (**Supplementary Figures S3C, D**). Representative markers for each cell type are shown (**Figure 4C**; **Supplementary Figure S3E**). Single-cell sequencing revealed transcriptome differences for each cell type between tumors and normal tissues (**Figure 4D**). The results show that macrophages, T cells, and epithelial cells are notably enriched in tumor tissues, while other cells are highly represented in normal tissues.

Next, MDLS was integrated into the single-cell analysis for scoring (**Figure 4E**). The cells were segregated into two groups based on the media MDLS scores of the epithelial cells (**Figure 4F**). The potential pathways of MDLS were enriched and visualized by differential expression analysis and GSEA (**Supplementary Figures S3F, G**). A tumor microenvironment consists of tumor epithelial cells, tumor stromal cells, and extracellular matrix. Most cancers are caused by a destroyed epithelial cell population, causing tumor cells to grow rapidly. Take the epithelial cells for example, high-MDLS group was notably enriched in proteasome, focal adhesion, Ribosome, spliceosome. While the low-MDLS group was predominantly associated with reactive oxygen, oxidative phosphorylation (**Supplementary Figure S3G**). Further observation on copy-number alteration by the copyKat algorithm was employed to distinguish between normal cells and tumor cells (**Figure 4G**). Ultimately, a higher MDLS score was observed in tumor-aneuploid cells compared to tumor-diploid cells, indicating the significance of MDLS in breast cancer progression (**Figure 4H**).

### Exploring specific regulatory factors driving MDLS and cell recognition

**Figure 5:**
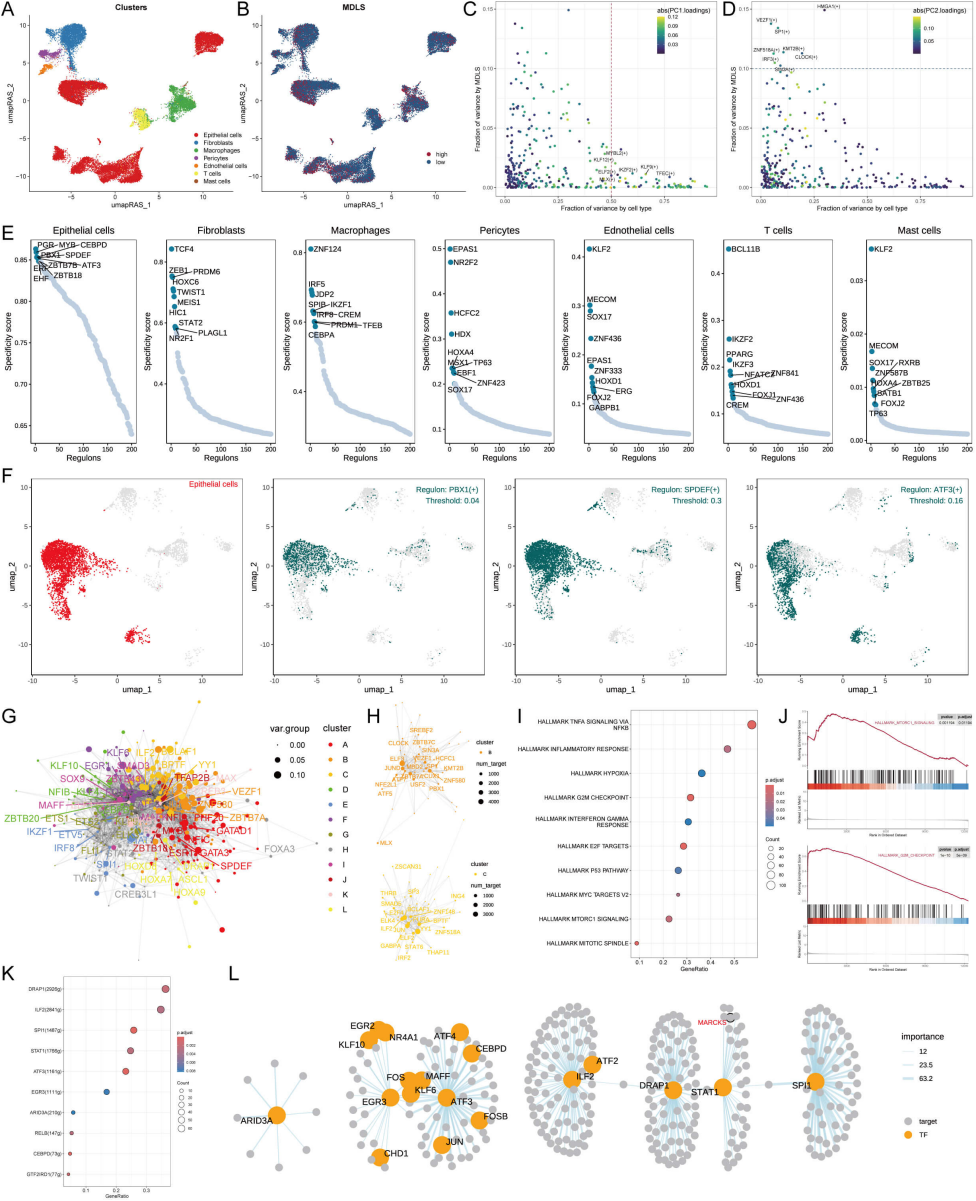
Exploring specific regulatory factors driving MDLS and cell recognition. **(A)** Distinct clusters within a cell population based on RAS. **(B)** MDLS levels across the cell population, with varying color intensities reflecting the magnitude of scores. **(C)** A variance analysis plot highlights the impact of cell types and MDLS on transcription factor activity, using color mapping to PC1 to emphasize the primary variance influenced by these factors. **(D)** Variance analysis plot, color mapped to PC2, explores additional dimensions of MDLS. **(E)** Key regulators for 7 cell types, and specific scores for each regulator. **(F)** The most specific regulator in epithelial cells (PBX1, SPDEF and ATF3). **(G)** The network graph using the Leiden algorithm, mapped. **(H)** The graph concentrates on modules A and D, which significantly contribute to MDLS. **(I)** GSEA identifies pathway variations linked to MDLS in epithelial cells. **(J)** Representative pathways activated or inhibited in the context of high MDLS. **(K)** Transcription factors contributing to the activation pathway. **(L)** The regulatory networks of mtorc1 signaling.

We used the SCENIC pipeline to analyze single-cell RNA-seq data with cis-regulatory sequence information to comprehensively construct GRNs. The UMAP analysis visualized the cell data into seven cell clusters and performed RAS scores on the MDLS score (**Figures 5A, B**). In summary, the gene expression information was converted to the activity score of transcription factors known as RAS. PCA and variance analyses revealed the transcription factors associated with the formation of MDLS and cell types (**Figures 5C, D**).

We identified seven key regulatory factors for cell types and scored each regulator specificity according to Jensen-Shannon divergence. The regulator with higher regulon specificity score (RSS) might be correlated specifically with that cell type (**Figure 5E**). We selected the regulator with the highest RSS value for each cell type to further examine its functional properties (**Figure 5F**; **Supplementary Figure S4A**). Take the epithelial cells for example, PBX1, SPDEF and ATF3 were found to be the most specific regulatory factors in the RSS sequencing of epithelial cells (**Figure 5F**).

Each cell type has its own form and function, and the characteristics of the cell type need to be maintained by the coordinated interaction of transcription factors and their corresponding target genes. According to the Leiden algorithm, we compared the RAS scores of each regulon pair across the atlas to characterize the combinatorial patterns of the MDLS. A highly modular diagram shows the formation of 12 modules (**Figure 5G**; **Supplementary Figure S4B**). The module B and C play a key role in MDLS progression (**Figure 5H**; **Supplementary Figure S4B**). We focus on the key transcription factors that drive epithelial cell transcriptome changes in MDLS. Using GSEA analysis to identify multiple pathway variants in epithelial cells, MAPK/MTORC1 signaling and G2M checkpoint were activated in the high-MDLS group (**Figures 5I, J**). The results further confirmed that transcription factors are involved in these pathways and the progression of MDLS (**Figure 5K**). The detailed regulatory network of transcription factors that influence MDLS progression were further demonstrated (**Figure 5L**).

### Cell-cell communication based on MDLS

**Figure 6:**
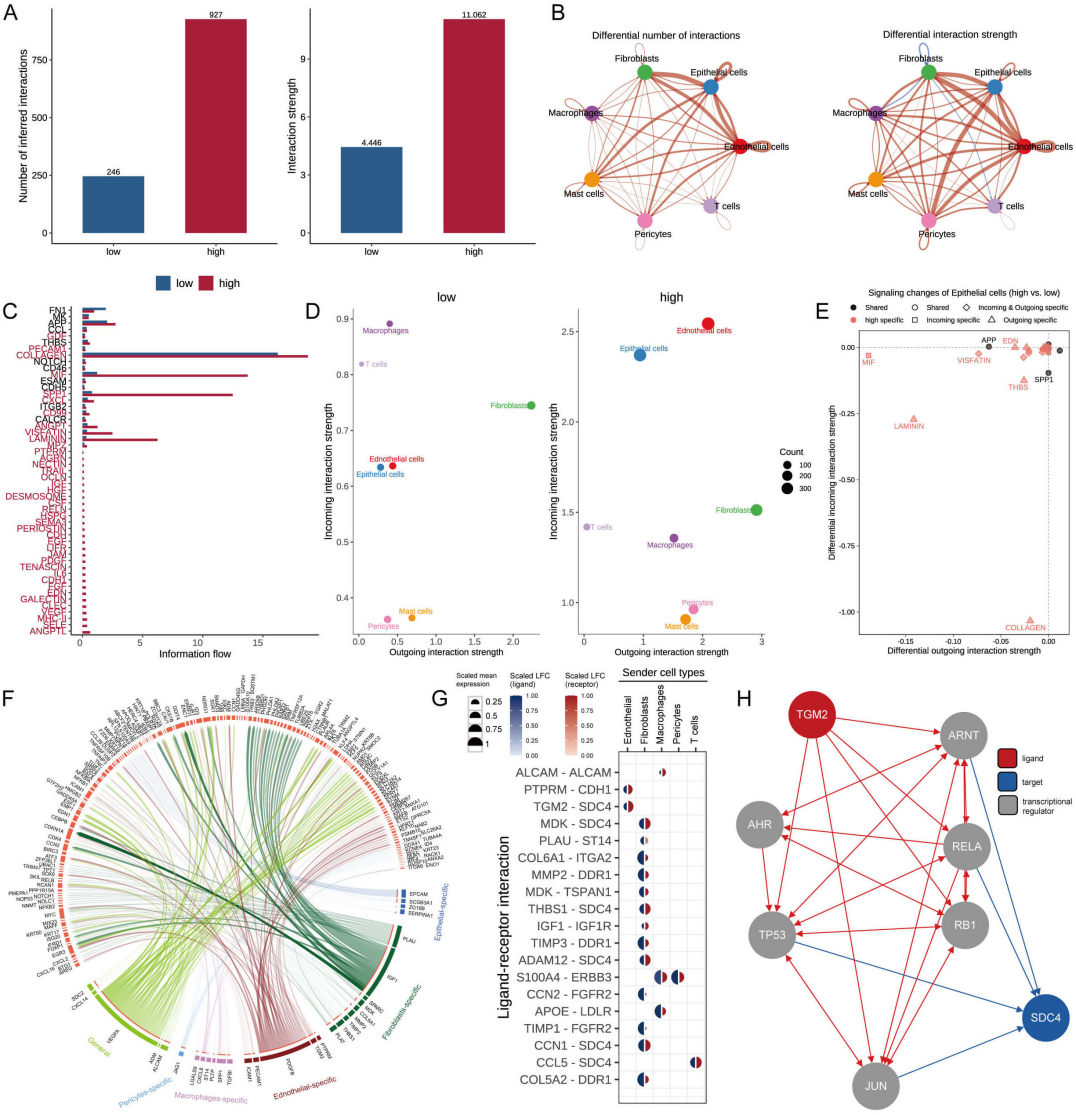
Cell-cell communication based on MDLS. **(A)** Number and strength of cellular interactions in MDLS groups. **(B)** The number and intensity of intercellular communication in 7 cell types. **(C)** The signal pathways involved in intercellular communication of MDLS. **(D)** Scatter plots compare outgoing and incoming interaction strengths between cell types in low and high MDLS. **(E)** Pathway specificity in epithelial cells includes notably specific pathways in aggressive cancer phenotypes. **(F)** Ligand-receptor pair interactions in different cell types. **(G)** Top-predicted ligand-receptor pairs, pointing to heightened interactions, especially involving TGM2-SDC4 in high MDLS cells, indicative of aggressive behavior. **(H)** A detailed map of the routes of TGM2 ligands to the target receptor SDC4.

To emphasize the complex interactions between cells interactions in BC progression, we applied the CellChat analysis to assess communication between seven different types based on MDLS. The number and strength of interactions were assessed, revealing that the group with high-MDLS had higher cell-to-cell communication (**Figure 6A**). In the high-MDLS group, endothelial cells, epithelial cells, and fibroblasts displayed a massive amount of interaction. Nevertheless, macrophages and T cells have weaker interactions with other cells (**Figure 6B**). We further investigated 51 signaling pathways of cell communications, therein, 48 signaling pathways were primarily activated in high-MDLS cells, including laminin, collagen, and cxcl, except for FN1, MK, and CCL (**Figure 6C**). The interaction between endothelial and epithelial cells in the high-MDLS group was stronger, while the communication with T cells was weaker (**Figure 6D**). Using epithelial cells as an example, the high-MDLS group had higher pathway specificity compared to the low-MDLS group, such as MIF, VISFATIN, LAMININ and THBS pathway (**Figure 6E**).

Nichenetr analysis was utilized to assess the activity of ligands regulating epithelial cell incoming and outgoing in different MDLS groups. Further analyses were focused on the differences in the activity of the ligand-receptor pairs. A Circos diagram illustrates the interaction of ligand-receptor pairs in different cell types (**Figure 6F**). The high interaction of TGM2-SDC4 indicates that fibroblasts and endothelial cells are the primary transmitter cells influencing changes in the epithelial cell pathway (**Figure 6G**). **Figure 6H** provides a detailed roadmap of TGM2 ligand reaching the target receptor SDC4 through other receptors or transcription factors.

### Analyzing potential immunotherapy targets for MDLS

**Figure 7:**
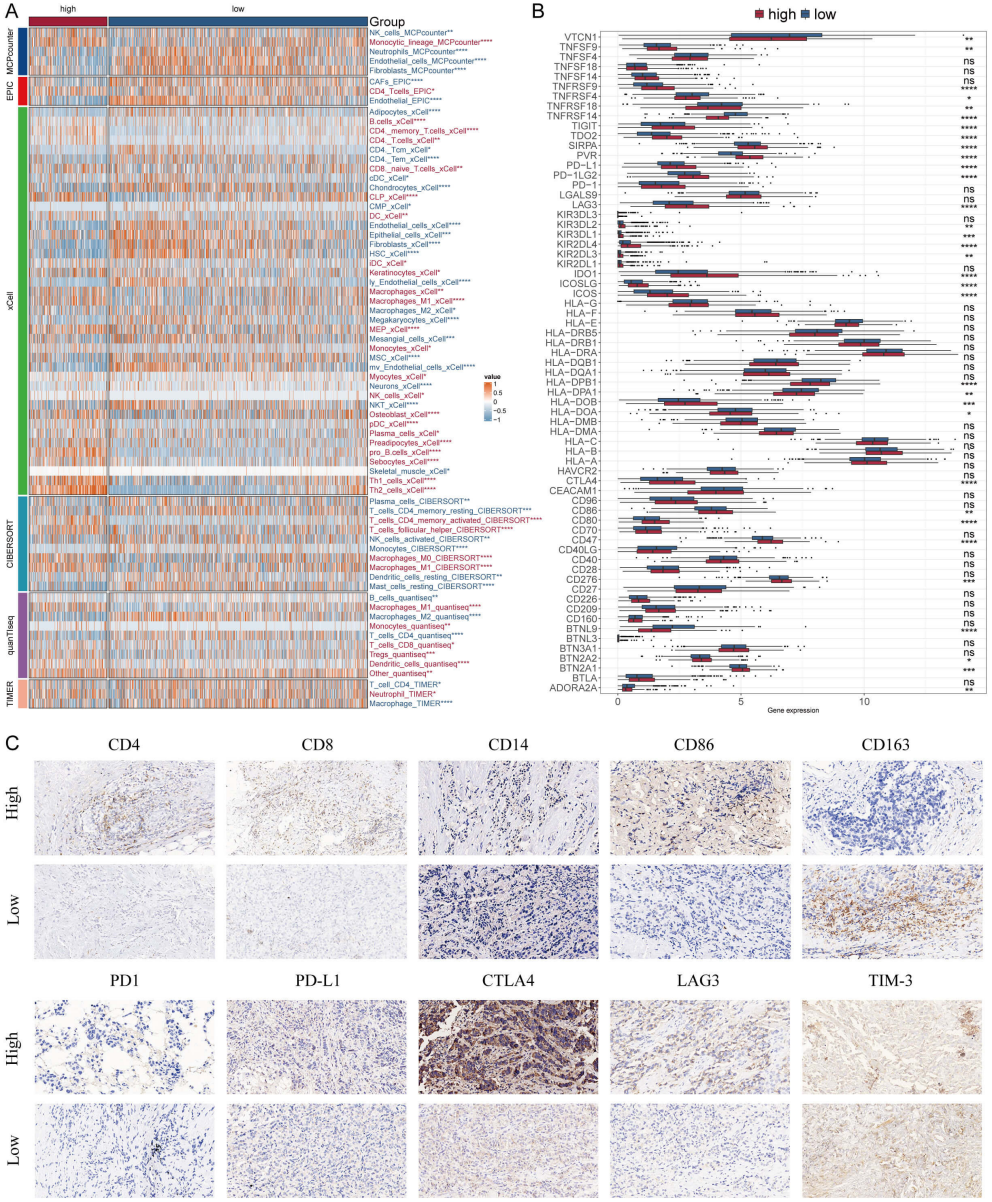
Differential expression and immunohistochemical analysis of immune markers in tumor microenvironments between MDLS subgroups. **(A)** Heatmap providing a comparative view of immune cell infiltration in tumor samples with low and high MDLS, utilizing various computational algorithms for quantification. Each row represents a different type of immune cell, with the color intensity reflecting the level of infiltration. Red text indicates increased infiltration in the high MDLS group, while blue text indicates decreased infiltration. **(B)** Box plots illustrating the distribution of gene expression levels for ICIs across low versus high MDLS conditions, with statistical significance denoted by ns for not significant; *P < 0.05; **P < 0.01; ***P < 0.001; ****P < 0.0001. **(C)** Representative immunohistochemistry images showcasing the staining intensity of various immune markers between high and low expression conditions, visually depicting the differential expression of these markers in correlation with MDLS levels.

We applied six algorithms to evaluate the immune infiltration. High-MDLS group had more immune infiltration, such as CD4^+^ memory T cells, CD8^+^ T cells and M1 macrophages (**Figure 7A**). The expression of ICIs is also assessed as a key indicator of immunotherapy responsiveness. The expression of ICIs was higher in the high-MDLS group, such as TNFRSF14, PD-1, PD-L1, LAG3, IDO1 (**Figure 7B**). IHC was performed to support the above results using the representative cell markers and clinical ICIs (**Figure 7C**).

**Figure 8:**
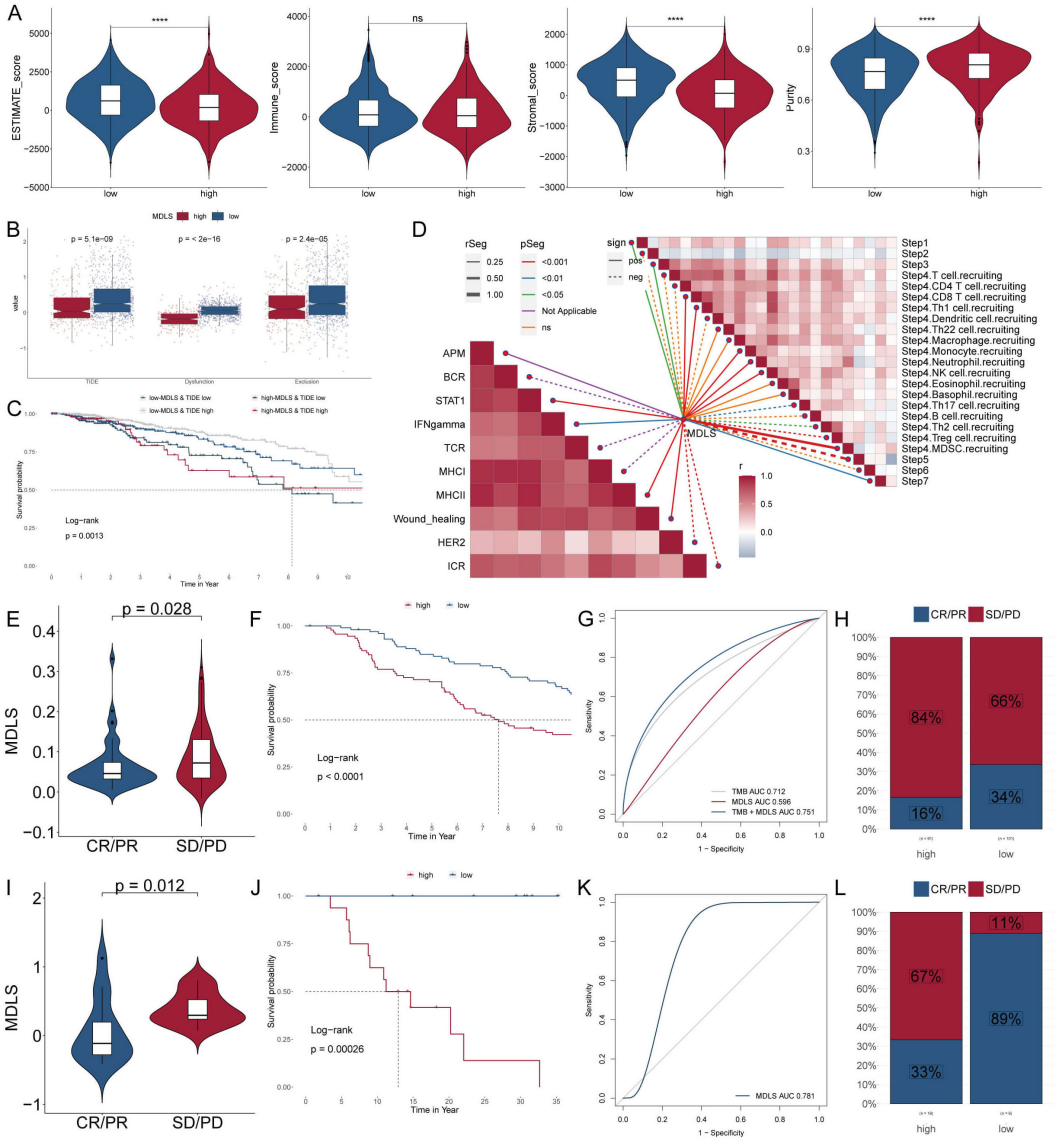
Analyzing potential immunotherapy targets for MDLS. **(A)** ESTIMATE scores, immune score, stromal scores and tumor purity between tow MDLS patients. **(B)** Difference of TIDE, Dysfunction, Exclusion between the MDLS groups. **(C)** The survival probability curves of four combinations of MDLS and TIDE. **(D)** The correlation of MDLS with 7 steps of tumor immune cycle and 10 signaling pathways related to tumor immunology. **(E, I)** Violin charts display the relationship between MDLS levels and responses to anti-PDL1 **(E)** and anti-PD1 **(I)** therapies. **(F, J)** Survival probabilities of low and high MDLS patients in anti-PDL1 **(F)** and anti-PD1 **(J)** cohorts, respectively, illustrating the impact of MDLS on survival outcomes. **(G, K)** Analysis estimates the predictive ability of MDLS via AUC values, considering TMB combinations, in anti-PDL1 **(G)** and anti-PD1 **(K)** cohorts, evaluating the efficacy of MDLS as a biomarker. **(H, L)** The percentages of complete response/partial response (CR/PR) and stable disease/progressive disease (SD/PD) in anti-PDL1 **(H)** and anti-PD1 **(L)** cohorts are shown, based on MDLS levels, to assess treatment effectiveness. ****P < 0.0001.

Next, we used ESTIMATE analysis to predict tumor purity and the presence of infiltrating stroma/immune cells in tumor tissue. The results showed that low-MDLS patients had higher ESTIMATE scores and Stromal scores compared to the higher score patients but had lower tumor purity (**Figure 8A**). It suggests that the low-MDLS patients are more likely to receive immunotherapy. Meanwhile, TIDE value, dysfunction and exclusion value is higher in low-MDLS patients (**Figure 8B**). Notably, patients with low-MDLS combined with high-TIDE had a higher survival rate than patients with other types (**Figure 8C**). The results showed that the anti-tumor immune activity of low-MDLS patients was higher than that of high-MDLS patients (**Figure 8D**). Immune checkpoints have long been used in the immunotherapy of cancer, so we evaluated the ability of the MDLS to predict the immune checkpoint blocking response. MDLS in the IMvigor210 (anti-PD-L1) and GSE78220 (anti-PD-1) cohorts was further assessed. In IMvigor210 (**Figures 8E–H**) and GSE78220 (**Figures 8I–L**), patients with low-MDLS exhibited better survival rates and clinical benefits compared to those with high-MDLS. In summary, patients with low-MDLS may derive greater benefits from ICIs treatment.

### Identifying therapeutic agents for high-MDLS patients

**Figure 9:**
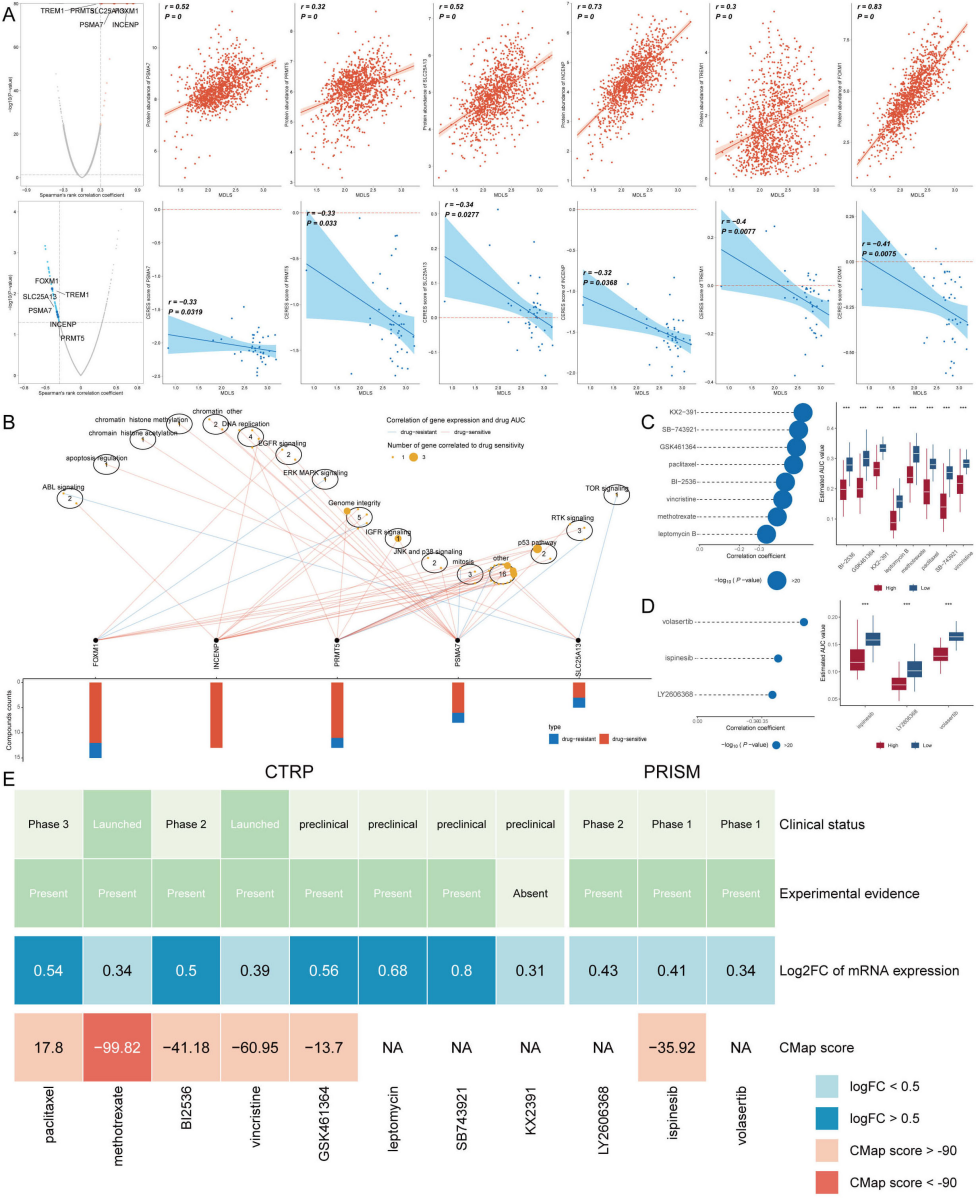
Identifying therapeutic agents for high-MDLS patients. **(A)** Spearman correlation of MDLS with 6 potential therapeutic targets expression and CERES value (red: positive correlation, blue: negative correlation). **(B)** Network analysis highlights the intricate connections between these five therapeutic targets and their associated drug action pathways. **(C, D)** Box plots compare the AUC values of identified compounds, sourced from the CTRP **(C)** and PRISM **(D)** datasets, between low and high MDLS patient groups. Observations of higher AUC values in low MDLS patients indicate less favorable chemotherapy outcomes for this subgroup, pointing to the need for personalized treatment strategies. **(E)** A summary table outlines the multi-perspective analysis of the nine candidate compounds, detailing their clinical status, experimental evidence, mRNA expression levels, and CMap scores. Vincristine and gemcitabine are highlighted as a potentially suitable therapeutic agent for high MDLS patients based on its favorable CMap score, suggesting it could be particularly effective in this patient subset. ^***^p<0.001.

Chemotherapy remains a cornerstone treatment for cancer. In this study, we devised a targeted approach for breast cancer patients with high-MDLS levels, leveraging sensitivity data collected from multiple datasets. Initially, we employed Spearman’s correlation analysis to identify key therapeutic targets. The analysis revealed a positive correlation between MDLS and the abundance of six potential targets (PSMA7, PRMT5, SLC25A13, INCENP, TREM1, FOXM1). Importantly, these targets also exhibited notably negative correlations with their respective CERES scores, indicating their potential as therapeutic targets for patients with high-MDLS levels (**Figure 9A**). Therein, five of six targets were found to be closely linked to various drug action pathways, underscoring their significance as critical therapeutic targets for this patient subgroup (**Figure 9B**).

Subsequently, we obtained 12 chemical compounds from the CTPR and PRISM datasets, including. An analysis comparing the AUC values of these compounds between the two MDLS groups revealed higher AUC values in patients with low-MDLS, indicating a less favorable response to chemotherapy in this demographic (**Figures 9C, D**). A comprehensive multiple-perspective analysis was then conducted to select the most effective therapeutic drugs from these 12 candidates. This analysis included detailed evaluations of each compound’s clinical status, experimental evidence, mRNA expression levels, and CMap scores. Ultimately, methotrexate was identified as the most suitable therapeutic drugs for patients with high-MDLS, based on their CMap score (**Figure 9E**).

## Discussion

The National Cancer Report 2019 states that breast cancer is now the predominant form of tumor among females, resulting in over 300,000 new cases and more than 66,000 fatalities annually (37). At present, chemotherapy for breast cancer still lacks effective molecular targeted therapy strategies. And low-sensitivity chemotherapy can easily cause drug resistance, reduce chemotherapy’s benefits, and lead to recurrences and metastases (38). Clinicians and researchers lack biomarkers for screening, stratification, and prognostic follow-up, leading to overtreatment and undertreatment. Improving the therapeutic effect has become the primary goal in the treatment of breast cancer, and one of the ways to achieve this goal is to construct an effective prognostic model of breast cancer.

In physics and chemistry, LLPS was originally defined as a technique for separating liquids from liquids (39). There has been preliminary evidence that LLPS plays a role in cell biology and oncology in recent years. A membranelles aggregate is formed in cells when the LLPS protein is activated (40). There are many normal physiological processes that are mediated by LLPS, including protein degradation, transcription, and DNA damage repair (41). As LLPS interacts with extracellular matrix, it may cause some carcinogenic condensates to form, activating downstream signaling pathways in tumor cells (42). Research has consistently demonstrated that genetic abnormalities are closely associated with cancer development, and phase separation can be a contributor to tumor growth (43). LLPS especially affects epigenetic dysregulation, which might trigger tumorigenesis and progression (44, 45). There is evidence that LLPS has therapeutic potential as a novel cancer intervention target (44). Hence, in this study, we constructed a prognostic model using LLPS genes to improve the prognosis of BC.

We first identified the differences in the roles of 52 LLPS genes across different datasets and pointed out the complex relationships between these genes and BC prognosis. The RSF algorithm was used to recruit key LLPS genes, and finally identified four genes (POP1, TUBA1C, PACGAP1 and PLK1) to build a prediction model.

Nucleus-localized POP1 encodes a ribonuclease involved in tRNA preprocessing (46). In patients with connective tissue disease, POP1 is also an autoantigen and suppresses inflammation (47, 48). In recent years, the potential of POP1 for the prognostic and diagnostic value of tumors has been preliminarily elucidated. Zhu et al. established a prognostic model for colorectal cancer, and POP1 is a new prognostic marker for colorectal cancer (49). Through bioinformatics analysis, Liang et al. found that POP1 was a gene in the pyroptosis-related prognostic model of gastric cancer (50). The TUBA1C subtype of α-tubulin is microtubule-related. It is a multifunctional cytoskeletal protein that plays an essential role in cell mitosis and cell division (51, 52). Studies have shown that when TUBA1C expression level is increased, the growth and progression of tumor cells are significantly affected (53, 54). In recent years, studies have also reported the potential role of TUBA1C in the immune system, including innate and adaptive immunity (55). Moreover, TUBA1C are found to be involved in the growth, invasion, and metastasis of lung cancer (56, 57). The RACGAP1 GTPase regulator mediates cytokinesis by activating RHOA and inactivating RAC1. The RacGAP1 receptor mediates the switching from Rac to RhoA activation that regulates cell motility and migration (58). An inhibition of migration and invasion is observed when RACGAP1 is silenced in cell lines that express it endogenously (59). Also involved in regulating cell proliferation is RACGAP1, which interferes with the mitotic spindle apparatus (60). Pol-like kinases (PLKs) are a kinase-like protein family with highly conserved structural domains that regulate cell cycle progression, and a main subtype of PLKs is Polo-like kinase 1 (PLK1) (61). The PLK1 protein is required for spindle assembly, mitosis, and DNA damage response as well as maintaining genomic stability (62). Tumors have been found to express PLK1 abnormally in numerous studies, including colorectal cancer (63), melanoma (64), cervical cancer (65). According to some studies, inhibiting the expression of PLK1 by antibodies, RNA interference, or kinase inhibitors is effective in inhibiting tumor cell proliferation and inducing cell death (66). Blocking the expression of PLK1 can result in the death of cancer cells by disrupting various phases of cell division, making PLK1 a promising candidate for cancer treatment (67).

Some types of solid tumors can be treated with cancer immunotherapies, but tumor cells employ camouflage and evolve to escape immune attack. Consequently, identifying effective biomarkers is essential to improving the efficacy of cancer treatments and predicting survival. According to the correlation between MDLS and immune infiltration, macrophage abundance is higher in breast cancer patients. It is believed that monocyte-attracting chemokines are primarily responsible for macrophage infiltration in tumors, such as CCL2 and CCL5 that can be produced by tumor cells, endothelial cells, macrophages, and fibroblasts within the tumor microenvironment (68). It has been reported that tumor-infiltrating macrophages frequently have a more “tumor-promoting” M2 phenotype as a result of exposure to Th2 cytokines such as IL-13 and IL-4, and the immunosuppressive cytokines TGF-β and IL-10 (69). The M1 (classical macrophage phenotype) macrophage, in contrast, can develop anti-tumor properties when stimulated by antimicrobial products such as lipopolysaccharide with or without concurrent exposure to proinflammatory cytokines (70). Reports on human and canine mammary carcinomas indicate that macrophage infiltration is related to poorer prognoses, despite the diversity among macrophage subsets (71). It is speculated that high levels of M2 macrophage infiltration are associated with poor prognosis of breast cancer.

Subsequently, transcriptome analysis identified the MDLS activity of eight types of immune cells at the single-cell level and found that the MDLS activity of tumor aneuploid epithelial cells was higher than that of tumor diploid and normal samples. It is believed that epithelial-mesenchymal transitions (EMT) are responsible for distant metastases from epithelial cancers like breast cancer. In EMT, intercellular tight junctions are disrupted, and the cell-cell connection is lost. The morphology of the epithelium is reduced and the mesenchyme is gained as a result (72). Self-renewal of these cells is increased, as well as heterogeneity in their subpopulations. Self-renewal of these cells is increased, as well as heterogeneity in their subpopulations. The gene map revealed that many genes are differentially expressed during EMT, and we identified several interrelated pathways and a set of signaling molecules involved in the EMT process and subsequent tumor metastasis and progression. CellChat analysis showed that cell-to-cell interactions and ligand-receptor interactions were stronger in the normal group. These results suggest that stronger intercellular communication is beneficial to the development of organisms.

This study revealed a counterintuitive yet significant finding: patients classified within the low- MDLS group exhibited a better response to immunotherapy despite having lower counts of CD8^+^ T cells and reduced expression of immune inhibitory factors compared to their high-MDLS counterparts. These results highlight the complexity of the TME and suggest that the mere quantitative presence of cytotoxic lymphocytes may not be the sole determinant of an effective anti-tumor immune response. The diminished expression of immune inhibitory checkpoints in the low-MDLS group, such as PD-1, CTLA-4, and PD-L1, observed in our data, suggests a less suppressive TME. Typically, these inhibitory molecules play pivotal roles in immune escape mechanisms by hindering T-cell effector functions. Lower levels of these inhibitors might, therefore, imply a TME that is less adept at evading immune surveillance, thereby facilitating a more robust immune-mediated tumor rejection despite the numerically lower presence of CD8^+^ T cells.

Furthermore, the functional quality and the interplay of immune cells within the TME might provide additional insight. Even with fewer CD8^+^ T cells, the immunological milieu in low-MDLS patients might be characterized by a higher proportion of functionally potent and less exhausted T cells. This hypothesis aligns with recent studies suggesting that the activation state and functional capacity of T cells can be more critical than their absolute numbers in determining the outcome of cancer immunotherapy. Additionally, the orchestration of various immune cells, including regulatory T cells, myeloid-derived suppressor cells, and other components of the immune cell repertoire, could differ fundamentally between the two groups, influencing the overall treatment response. The lower expression of immune inhibitory factors in the low-MDLS group may also facilitate a more effective antigen presentation and T-cell priming, further enhancing the anti-tumor immune response.

The findings from this study advocate for a more nuanced understanding of the TME and suggest that the interrelationships and functional states of different immune components can critically influence the efficacy of immunotherapy. They also underscore the potential of integrating comprehensive immune profiling into clinical decision-making to tailor immunotherapeutic strategies more precisely.

In conclusion, the differential response to immunotherapy in breast cancer groups underscores the importance of considering qualitative and functional aspects of the immune cells, beyond their numerical abundance. This approach could lead to more personalized and effective therapeutic interventions, particularly in immunotherapy.

## Data Availability

The original contributions presented in the study are included in the article/**Supplementary Material**. Further inquiries can be directed to the corresponding authors.
