# Supplementary material for: Machine learning-informed liquid-liquid phase separation for personalized breast cancer treatment assessment
Source: Front Immunol. 2024 Nov 19;15:1485123. doi: 10.3389/fimmu.2024.1485123 (PMC11611825; doi:10.3389/fimmu.2024.1485123)
Supplement: Supplementary file 1 [file DataSheet1.pdf]

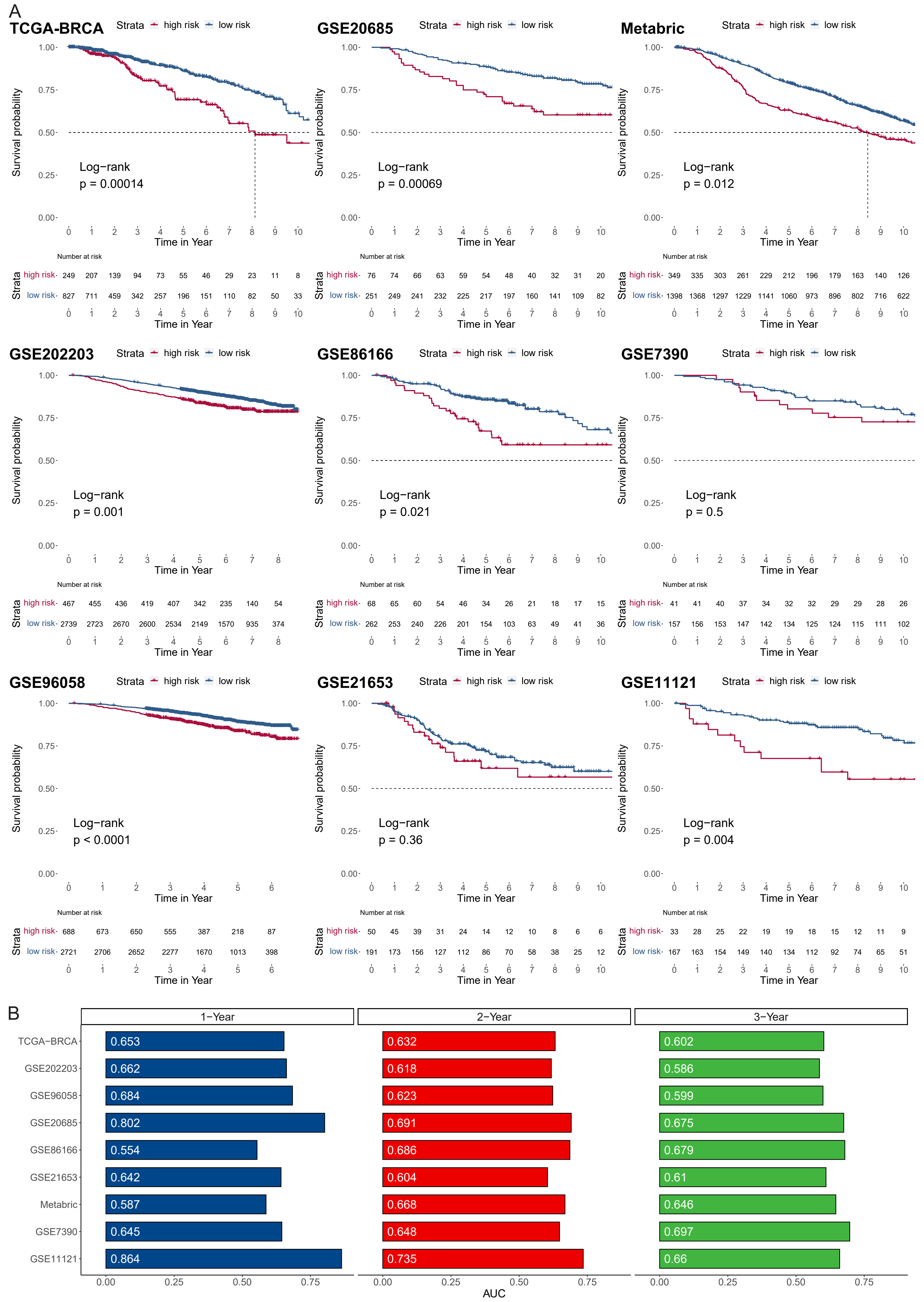

Figure S1. Evaluation of MDLS in 9 cohorts. (A) Kaplan-Meier curves of the MDLS in 9 cohorts. (B) Time-dependent ROC analysis for predicting OS at 1, 3, and 5 years.
