# Supplementary material for: Machine learning-informed liquid-liquid phase separation for personalized breast cancer treatment assessment
Source: Front Immunol. 2024 Nov 19;15:1485123. doi: 10.3389/fimmu.2024.1485123 (PMC11611825; doi:10.3389/fimmu.2024.1485123)
Supplement: Supplementary file 3 [file DataSheet3.pdf]

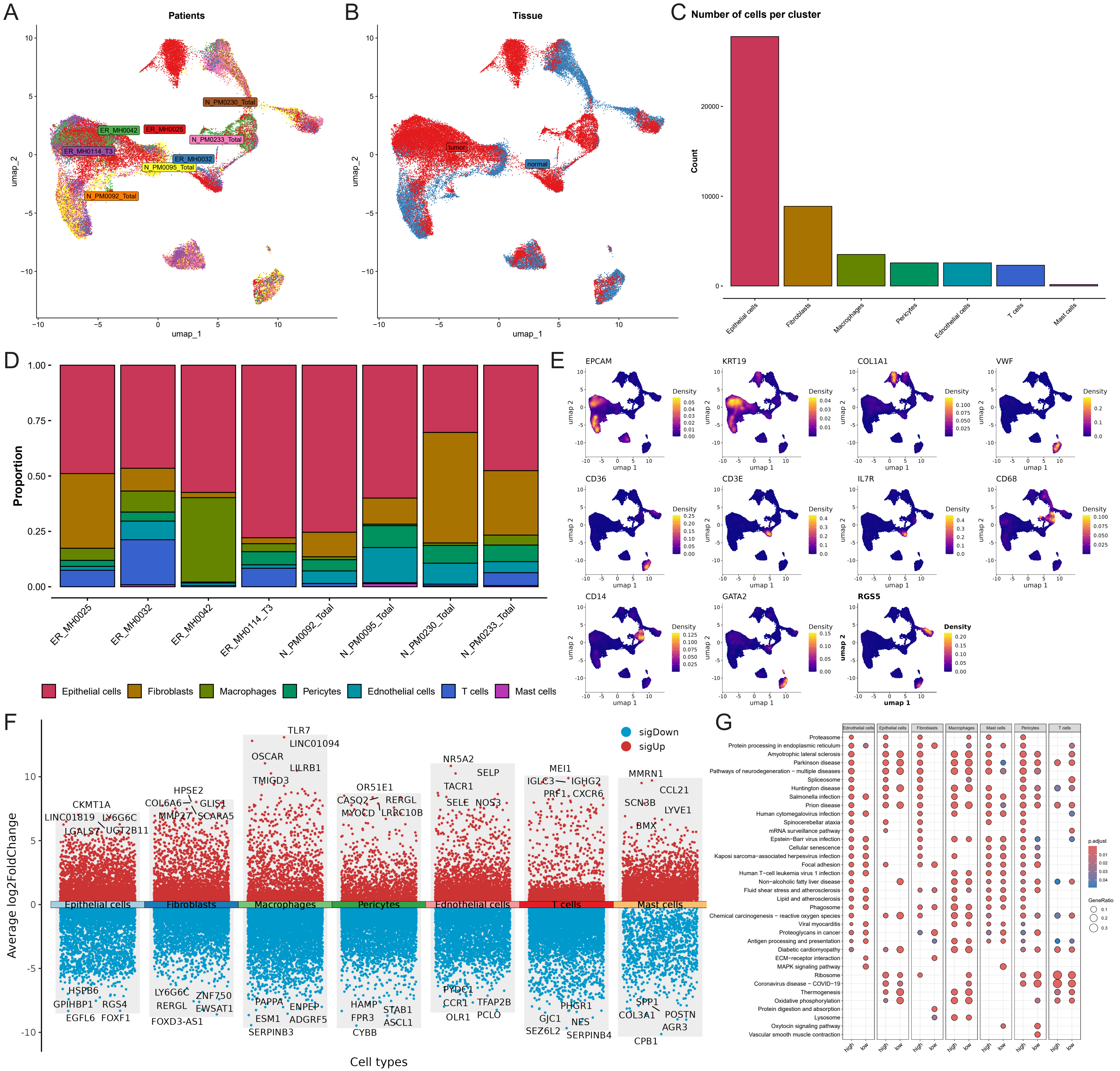

Figure S3. Comprehensive single-cell profiling of breast cancer. (A) A UMAP visualization illustrates the distribution of single cells from multiple breast cancer patients. (B) UMAP plot categorizes tissue type, with red dots representing tumor cells and blue dots indicating cells from normal tissue. (C) A bar graph displays the number of cells per cluster identified in the dataset, with the largest bar representing epithelial cells, indicating the prevalence of this cell type in the samples. (D) A stacked bar chart shows the proportion of different cell types found in individual patients, illustrating the variability in cellular composition between patients. (E) UMAP plots each highlight the density of specific marker genes across cells, providing insights into the expression patterns that may define cell function or status. (F) A volcano plot illustrates differential gene expression across specific cell types, with genes that are significantly upregulated shown in red and downregulated in blue. Labels identify some of the most differentially expressed genes, offering clues to their potential roles in cancer biology. (G) A dot plot matrix reveals the probable involvement in specific biological processes of genes that are differentially expressed among each cell type, linking gene expression patterns to potential functional implications.
