# Supplementary material for: Machine learning-informed liquid-liquid phase separation for personalized breast cancer treatment assessment
Source: Front Immunol. 2024 Nov 19;15:1485123. doi: 10.3389/fimmu.2024.1485123 (PMC11611825; doi:10.3389/fimmu.2024.1485123)
Supplement: Supplementary file 4 [file DataSheet4.pdf]

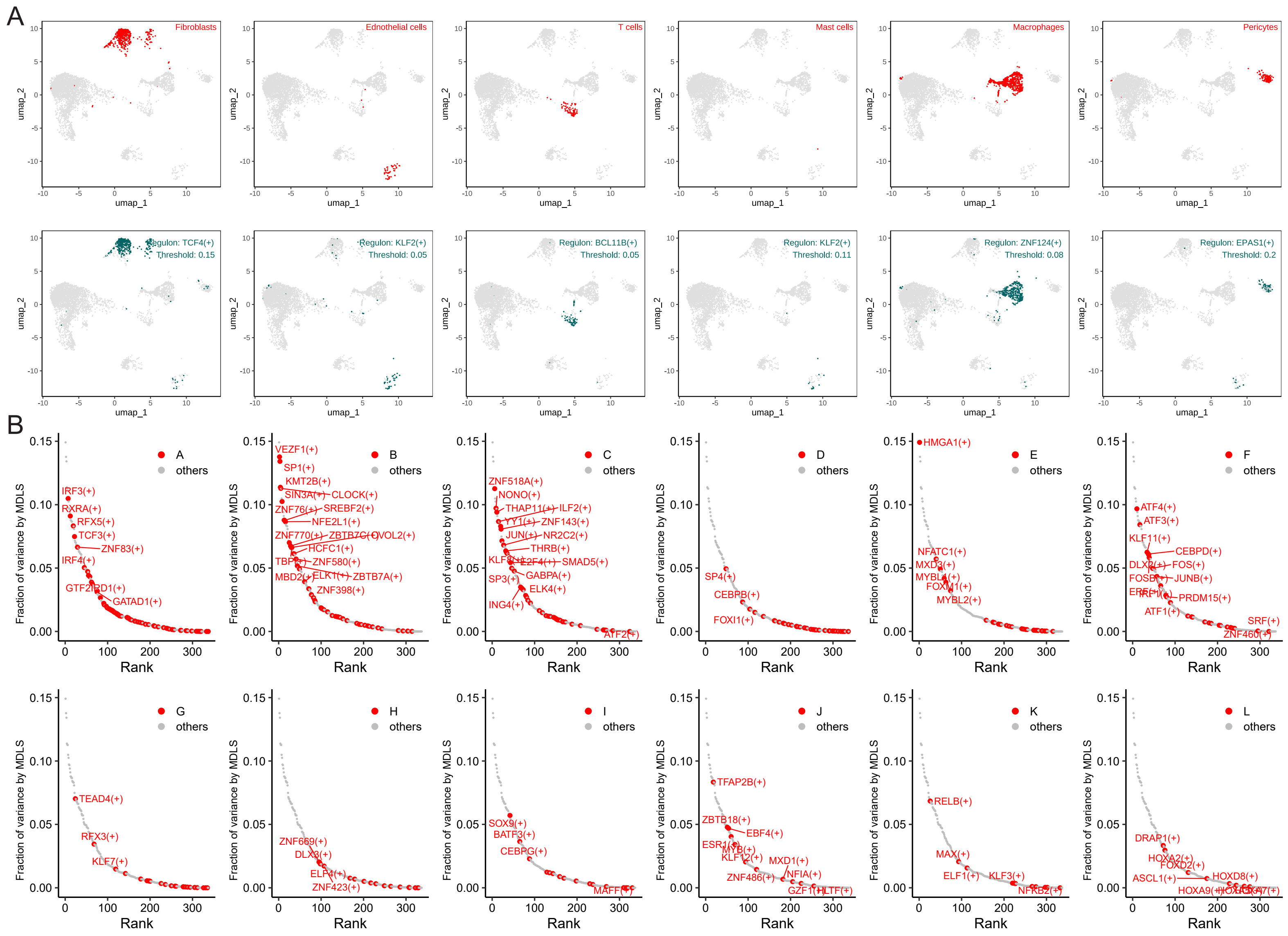

Figure S4. Transcription factor activity correlation and contribution analysis in cell types. (A) UMAP projections categorize samples by transcription factor activity, revealing distinct cell types based on their transcriptional profiles, aiding in the identification of unique cellular behaviors and properties. (B) Illustrates the contribution of different transcription factor groups to MDLS, with significant transcription factors highlighted and ranked based on their RSS.
